# Complete characterization of anisotropic geodesics in the Euclidean space

**DOI:** 10.1007/s10711-026-01111-z

**Published:** 2026-09-10

**Authors:** Pietro Aldrigo

**Affiliations:** Mathematisches Institut (MAI), Universität Bern, Sidlerstrasse 12, 3012 Bern, Switzerland

**Keywords:** Geodesics, Anisotropic energies, Isoperimetric sets, Primary 49K21, 49Q10, 51M25

## Abstract

We provide a characterization of absolutely continuous paths that minimize the anisotropic *F*–length between two points, where *F* is a lower semi–continuous, 1–homogeneous positive function defined on ${\mathbb {R}^{n}}$. The characterization is achieved by establishing a connection between the minimizing paths and the geometry of the anisotropic *F*–isoperimetric set.

## Introduction and main results

The study of geodesics has a rich history in several areas of mathematics (see e.g. [2, 10, 12, 15, 17]) and its applications range from path planning in robotics [23, 25] to image processing [7], and more. On the other hand, significant advances have been made in the study of the geometric properties of sets arising as critical points of anisotropic functionals (e.g. [3–6, 11, 14, 18, 20–22, 24]). The present work lies at the intersection of the two aforementioned fields. We present a complete characterization of anisotropic geodesics (definition given below) in the Euclidean space, achieved through the establishment and application of a connection between these geodesics and the geometric properties of anisotropic *F*–isoperimetric set.

### The *F*–geodesic problem

Throughout this work *n* is an integer greater or equal than 2. We denote by ${\mathbb {S}^{n-1}}\subseteq {\mathbb {R}^{n}}$ the (n-1)–dimensional unit sphere centered at the origin.

A function $F:{\mathbb {R}^{n}}\rightarrow {\mathbb {R}}$ is 1–homogeneous if $F(\lambda x)= \lambda F(x)$ for every $\lambda \ge 0$ and $x\in {\mathbb {R}^{n}}$. Observe that any 1–homogeneous function is univocally determined by its values on ${\mathbb {S}^{n-1}}$. We say that a 1–homogeneous function is positive if it is positive in ${\mathbb {S}^{n-1}}$. An *integrand* is a lower semicontinuous, 1–homogeneous positive function and the set of all integrands is denoted by $\mathbb {I}$.

Denote by $\text { AC}([0,1];{\mathbb {R}^{n}})$ the family of absolutely continuous functions $\gamma :[0,1]\rightarrow {\mathbb {R}^{n}}$. It is well–known that if $\gamma \in \text { AC}([0,1];{\mathbb {R}^{n}})$ then γ admits a derivative $\dot{\gamma }(t)$ at almost every t∈(0,1) and $t\mapsto \dot{\gamma }(t)$ belongs to $L^1([0,1])$. We say that γ is *regular* if $|\dot{\gamma }(t)|>0$ for almost every t∈(0,1).

#### Definition

*(Anisotropic* ${\boldsymbol{F}}$ *–length)* Let $\gamma \in \text { AC}([0,1];{\mathbb {R}^{n}})$ and let *F* be an integrand. The anisotropic *F*–length (or *F*–length) of γ is the quantity

$$\begin{aligned} {\mathfrak {L}_F}(\gamma ):= \int _0^1 F(\dot{\gamma }(t))\,dt. \end{aligned}$$

Observe that the definition of *F*–length is invariant under reparametrization of γ, i.e. if $\gamma ,\rho \in \text { AC}([0,1];{\mathbb {R}^{n}})$ and $\rho (t)=\gamma (\tau (t))$ for some strictly increasing absolutely continuous function $\tau :[0,1]\rightarrow [0,1]$ such that τ(0)=0 and τ(1)=1, then

$$\begin{aligned} {\mathfrak {L}_F}(\rho ) = \int _0^1 F(\dot{\gamma }(\tau (t)))\tau '(t)\,dt = \int _0^1 F(\dot{\gamma }(s))\,ds = {\mathfrak {L}_F}(\gamma ). \end{aligned}$$

Moreover, in the special case of $F\big |_{\mathbb {S}^{n-1}}\equiv 1$, the *F*–length of a curve $\gamma \in \text { AC}([0,1];{\mathbb {R}^{n}})$ coincides with the classical length of γ.

The *F*–geodesic problem associated to $(x,y)\in {\mathbb {R}^{n}}\times {\mathbb {R}^{n}}$ is the following:

GP $$\begin{aligned} \text {minimize} \quad {\mathfrak {L}_F}(\gamma )\quad \text {over}\quad \gamma \in \text { AC}([0,1];{\mathbb {R}^{n}})\text { s.t. }\gamma (0) = x \text { and }\gamma (1)=y. \end{aligned}$$

We call the solutions (if any) of the problem (GP) *F–geodesics from* *x* *to* *y*, and we collect all such solutions into the set $F\text { --Geo}(x,y)$. We say that $\gamma \in \text { AC}([0,1];{\mathbb {R}^{n}})$ is a *F–geodesic* and write $\gamma \in F\text { --Geo}$ if γ is an *F*–geodesic from γ(0) to γ(1).

### Main results

Let $F\in \mathbb {I}$ be a fixed integrand. For each $v\in {\mathbb {S}^{n-1}}$ consider the half–space

$$\begin{aligned} H_v:=\{x\in {\mathbb {R}^{n}}: \langle x,v\rangle \le F(v)\}. \end{aligned}$$

#### Definition

*(F* *–crystal)* The *F–crystal* is the convex set

$$\begin{aligned} K_F := \bigcap _{v\in {\mathbb {S}^{n-1}}} H_v. \end{aligned}$$

It turns out that, under the standing assumptions of *F*, $K_F$ is a compact set containing 0 in its interior. This set is also known in the literature as Wulff’s set and it enjoys the following anisotropic isoperimetric property. Let $\Omega \subseteq {\mathbb {R}^{n}}$ be a set of finite perimeter (see e.g. [9, Chapter 5] for the definition and main properties of these sets). The *F–perimeter of* Ω is defined as

$$\begin{aligned} \text {Per}_F(\partial \Omega ):= \int _{\partial \Omega } F(\nu _\Omega (x))\,d{\mathscr {H}^{n-1}}(x), \end{aligned}$$

where $\partial \Omega$ is the (reduced) boundary of Ω, $\nu _\Omega (x)$ is the outer unit normal to $\partial \Omega$ at *x* and ${\mathscr {H}^{n-1}}$ denotes the (n-1)–dimensional Hausdorff measure. Denoting by |·| the Lebesgue measure in ${\mathbb {R}^{n}}$ and setting $\omega _n:=|\{x\in {\mathbb {R}^{n}}: |x|\le 1\}|$, it turns out that

1 $$\begin{aligned} \frac{\text {Per}_F(\partial \Omega )}{|\Omega |^{\frac{n-1}{n}}} \ge \frac{\text {Per}_F(\partial K_F)}{|K_F|^\frac{n-1}{n}} = n \omega _n^\frac{1}{n} \end{aligned}$$

holds for every admissible $\Omega \subseteq {\mathbb {R}^{n}}$. Moreover, equality in (1) holds if and only if, up to sets of measure zero, Ω is homothetic to $K_F$ (see e.g. [8, 20–22, 24]).

For any subset $\Omega \subseteq {\mathbb {R}^{n}}$, the *polar body of* Ω is defined as

$$\begin{aligned} \mathfrak {P}\Omega :=\left\{ z\in {\mathbb {R}^{n}}: \langle z,x \rangle \le 1 \,\forall x\in \Omega \right\} . \end{aligned}$$

Since $\mathfrak {P}\Omega$ is the result of the intersections of the half-spaces $\left\{ z\in {\mathbb {R}^{n}}: \langle z,x\rangle \le 1\right\}$ for any $x\in \Omega$, then $\mathfrak {P}\Omega$ is a convex subset containing the origin. Moreover, if Ω is a convex subset containing 0, then $\mathfrak {P}\mathfrak {P}\Omega = \Omega$ (see Lemma 4).

If $\Omega \subseteq {\mathbb {R}^{n}}$ and $\alpha \ge 0$, the set $\alpha \Omega$ is the set containing all of the elements αx for each $x\in \Omega$. We define the function $\Vert \cdot \Vert _F:{\mathbb {R}^{n}}\rightarrow {\mathbb {R}_{\ge 0}}$ as

$$\begin{aligned} \Vert z \Vert _F := \min \{\lambda \ge 0 : z\in \lambda \mathfrak {P}K_F\}. \end{aligned}$$

Notice that $\Vert \cdot \Vert _F$ may fail to be a norm only because, in general, $\Vert -z \Vert _F\ne \Vert z \Vert _F$. For any $z\in {\mathbb {R}^{n}}\backslash \{0\}$, $z/\Vert z \Vert _F$ belongs to $\partial (\mathfrak {P}K_F)$. It is easy to show that for every $z\in {\mathbb {R}^{n}}$ we have

$$\begin{aligned} \left\langle z,x\right\rangle \le \Vert z \Vert _F \,\, \forall x\in K_F\quad \text {and}\quad \exists \overline{x}\in K_F \,\,:\,\, \left\langle z,\overline{x}\right\rangle = \Vert z \Vert _F. \end{aligned}$$

Given $F\in \mathbb {I}$, we define the *convex envelope of* *F* as 1–homogeneous positive the function $\mathcal {D}(F):{\mathbb {R}^{n}}\rightarrow {\mathbb {R}_{\ge 0}}$

$$\begin{aligned} \mathcal {D}(F)(x):= \sup _{v\in {\mathbb {S}^{n-1}}}\left\{ \inf _{\begin{array}{c} w\in {\mathbb {S}^{n-1}}\\ \langle x,w\rangle >0 \end{array}} \left\{ F(w)\frac{\langle v, x\rangle }{\langle v,w \rangle }\right\} \right\} . \end{aligned}$$

Observe that $\mathcal {D}(F)\le F$ for every $F\in \mathbb {I}$. The *contract set of* *F* is

$$\begin{aligned} \text { Cont}(F):=\left\{ x\in {\mathbb {R}^{n}}: F(x) = \mathcal {D}(F)(x)\right\} \end{aligned}$$

As both *F* and $\mathcal {D}(F)$ are 1–homogeneous, if $x\in \text { Cont}(F)$ then $\lambda x\in \text { Cont}(F)$ for every $\lambda \ge 0$ and $0\in \text { Cont}(F)$ for every integrand *F*. We say that $F\in \mathbb {I}$ is *convex* if $\text { Cont}(F)={\mathbb {R}^{n}}$.

Let $K\subseteq {\mathbb {R}^{n}}$ be a compact subset and fix $v\in {\mathbb {S}^{n-1}}$. The *supporting hyperplane of* *K* *associated with* *v* is the (affine) hyperplane $\pi _v$ such that

$$\begin{aligned} \pi _v = \left\{ z\in {\mathbb {R}^{n}}: \langle z,v\rangle = \alpha \right\} \quad \text {and}\quad \max _{y\in K}\left\{ \langle y,v\rangle \right\} = \alpha . \end{aligned}$$

The first main result of this work is the following characterization of the *F*–geodesics.

#### Theorem 1

Let $F\in \mathbb {I}$ be an integrand and $\gamma \in \text { AC}([0,1];{\mathbb {R}^{n}})$ be regular. Define $\widehat{v}:=\gamma (1)-\gamma (0)$ and let $\overline{x}\in K_F$ such that $\Vert \widehat{v} \Vert _F = \langle \widehat{v}, \overline{x}\rangle$. The following are equivalent:

1. γ is a *F*-geodesic;
2. $${\mathfrak {L}_F}(\gamma )=\Vert \widehat{v} \Vert _F$$;
3. for almost every t∈(0,1), $\dot{\gamma }(t)\in \text { Cont}(F)$ and $\overline{x} + (\dot{\gamma }(t))^\perp$ is a supporting hyperplane for $K_F$ at $\overline{x}$.

For any r>0 and $x\in {\mathbb {R}^{n}}$, we define the *F*–geodesic (closed) ball of center *x* and radius *r* as the set

$$\begin{aligned} \left\{ \gamma (1): \gamma \in F\text { --Geo},\,\gamma (0)=x, {\mathfrak {L}_F}(\gamma )\le r\right\} . \end{aligned}$$

Then, as an immediate consequence of Theorem 1, we deduce the following relation between the *F*–crystal and the *F*-geodesic balls.

#### Corollary 2

Let $F\in \mathbb {I}$ be an integrand. Then the *F*–crystal $K_F$ and the *F*–geodesic unitary ball centered at the origin are one the polar body of the other.

Using Theorem 1 together with some further remarks, we prove that if the integrand *F* is convex, then line segments are always *F*–geodesics (Corollary 8). However, as demonstrated in Example 9, it is possible that, even with a convex integrand *F*, line segments are not the sole *F*–geodesics.

#### Definition

*(Cone of normal directions)* Let $K\subseteq {\mathbb {R}^{n}}$ be any convex set. For each $y\in \partial K$, we define the *cone of normal directions of* ∂K *at* *y* as

$$\begin{aligned} \mathcal {N}_y\partial K := \left\{ v\in {\mathbb {R}^{n}}: \langle N, x-y\rangle \le 0 \quad \forall x\in K\right\} . \end{aligned}$$

Thanks to classical differentiability properties of convex functions, it turns out that $\mathcal {N}_y\partial K\cap {\mathbb {S}^{n-1}}$ is a singleton at ${\mathscr {H}^{n-1}}$–almost every point $y\in \partial K$. For any such points, we denote by $N_{\partial K}(y)$ the unique element of $\mathcal {N}_y\partial K\cap {\mathbb {S}^{n-1}}$ and we call it *(outer) unit normal to* ∂K *at* *y*. Whenever $N_{\partial K}(y)$ is defined,

$$\begin{aligned} (N_{\partial K}(y))^\perp := \left\{ z\in {\mathbb {R}^{n}}: \langle z, N_{\partial K}(y)\rangle = 0\right\} \end{aligned}$$

is the tangent space of ∂K at *y*. The *affine tangent space of* ∂K *at* *y* is given by $y + (N_{\partial K}(y))^\perp$. By convexity of *K*, it is easy to see that $y+(N_{\partial K}(y))^\perp = \pi _{N_{\partial K}(y)}$ at every $y\in \partial K$ such that $N_{\partial K}(y)$ is defined.

#### Definition

*(Set of orthogonal directions)* Let $K\subseteq {\mathbb {R}^{n}}$ be convex. The *set of orthogonal directions to* ∂K is

$$\begin{aligned} \text { Ort}(\partial K):= \left\{ v\in {\mathbb {R}^{n}}\backslash \{0\}: \exists y\in \partial K \text { s.t. } \exists N_{\partial K}(y)=\frac{v}{|v|}\right\} , \end{aligned}$$

Let $F\in \mathbb {I}$ and fix two distinct points $x,y\in {\mathbb {R}^{n}}$. We say that two curves $\gamma ,\rho \in F\text { --Geo}(x,y)$ are equivalent, and we write $\gamma \sim \rho$ if there exists an increasing reparametrization $\tau :[0,1]\rightarrow [0,1]$ of ρ such that $\gamma = \rho \circ \tau$. With ${F\text { --Geo}_{/\sim }}(x,y)$ we indicate the set of equivalence classes of $F\text { --Geo}(x,y)$ with respect to the equivalence relation ∼. The second main result is the following.

#### Theorem 3

Let $F\in \mathbb {I}$ and $x,y\in {\mathbb {R}^{n}}$ such that x≠y. Then the *F*–geodesic problem (GP) admits a solution. More precisely:

1. if $y-x\in \text { Ort}(\partial K_F)$, then ${F\text { --Geo}_{/\sim }}(x,y)$ contains one and only element, and a representative of it is the line segment t↦(1-t)x+ty.
2. if $y-x\not \in \text { Ort}(\partial K_F)$, then ${F\text { --Geo}_{/\sim }}(x,y)$ contains infinitely many elements.

## Preliminaries

### Definition

*(Convex hull)* Let $\Omega \subseteq {\mathbb {R}^{n}}$ be a subset. The convex hull of Ω is the set

$$\begin{aligned} [\Omega ]:=\left\{ (1-\lambda )x + \lambda y: x,y\in \Omega \right\} . \end{aligned}$$

Notice that the convex hull of a set Ω is the “smallest” convex set containing Ω, in the sense that if $U\supseteq \Omega$ is convex, then $U\supseteq [\Omega ]$.

### Lemma 4

For every $$\Omega \subseteq {\mathbb {R}^{n}}$$, $$\mathfrak {P}\mathfrak {P}\Omega = [\Omega \cup \{0\}]$$.

### Proof

First we prove that $[{\Omega }\cup \{0\}]\subseteq \mathfrak {P}\mathfrak {P}\Omega$. By virtue of the above remark, it is enough to show that ${\Omega }\subseteq \mathfrak {P}\mathfrak {P}\Omega$. Fix $x\in \Omega$. By definition of polar body then

$$\begin{aligned} \langle x,z\rangle \le 1\quad \forall z\in \mathfrak {P}\Omega . \end{aligned}$$

Therefore $$x\in \mathfrak {P}\mathfrak {P}\Omega $$.

For the converse inclusion, suppose the existence of a point $x\in (\mathfrak {P}\mathfrak {P}\Omega )\backslash [\Omega \cup \{0\}]$. Then there exists an affine hyperplane $\pi =\{x\in {\mathbb {R}^{n}}: \langle x,v\rangle = \alpha \}$, for some $v\in {\mathbb {S}^{n-1}}$ and $\alpha \ge 0$ separating the sets $\{x\}$ and $[\Omega \cup \{0\}]$, i.e.

2 $$\begin{aligned} \langle y,v\rangle < \alpha \quad \forall y\in [\Omega \cup \{0\}]\quad \text {and} \quad \langle x,v \rangle > \alpha . \end{aligned}$$

Since $0\in [\Omega \cup \{0\}]$, then α>0. Moreover, the first of (2) implies $v/\alpha \in \mathfrak {P}\Omega$. This, combined with the second of (2), contradicts the fact that $x\in \mathfrak {P}\mathfrak {P}\Omega$.

□

Define the operators $\mathcal {W},\mathcal {I},\mathcal {A},\mathcal {D}$ as

$$\begin{aligned} \mathcal {W}(F)(v):= \inf _{\begin{array}{c} w\in {\mathbb {S}^{n-1}}\\ \langle v,w\rangle >0 \end{array}}\left\{ \frac{F(w)}{\langle v, w\rangle }\right\} , \quad \mathcal {I}(F)(v):=\frac{1}{F(v)}, \\ \mathcal {A}(F)(v):= \sup _{w\in {\mathbb {S}^{n-1}}}\left\{ F(w)\langle v,w \rangle \right\} , \quad \mathcal {D}(F):={\mathcal {A}(\mathcal {W}(F))} \end{aligned}$$

for all $F\in \mathbb {I}$ and $v\in {\mathbb {S}^{n-1}}$, and extended by 1–homogeneity in ${\mathbb {R}^{n}}$. It is easy to see that the inequalities $\mathcal {W}(F)\le F$ and $F\le \mathcal {A}(F)$ hold true for every integrand *F*. Moreover, for any $v\in {\mathbb {S}^{n-1}}$ and $F\in \mathbb {I}$,

$$\begin{aligned} \mathcal {D}(F)(v) := \sup _{u\in {\mathbb {S}^{n-1}}}\left\{ \inf _{\begin{array}{c} w\in {\mathbb {S}^{n-1}}\\ \langle v,w\rangle >0 \end{array}}\left\{ F(w)\frac{\langle u,v\rangle }{\langle u,w\rangle }\right\} \right\} \le \sup _{u\in {\mathbb {S}^{n-1}}}\left\{ F(v)\right\} = F(v). \end{aligned}$$

Hence

3 $$\begin{aligned} \mathcal {D}(F)\le F\quad \forall F\in \mathbb {I}. \end{aligned}$$

If $F\in \mathbb {I}$, we call *polar graph of* *F* and *polar hypograph of* *F* the sets

$$\begin{aligned} \text { Graph}(F):=\{F(v)v\in {\mathbb {R}^{n}}: v\in {\mathbb {S}^{n-1}}\} = \{x\in {\mathbb {R}^{n}}: |x|= F(x/|x|)\},\\ \text { Hypo}(F):=\{\lambda F(v)v\in {\mathbb {R}^{n}}: v\in {\mathbb {S}^{n-1}},\,0\le \lambda \le 1\} = \{x\in {\mathbb {R}^{n}}: |x|\le F(x/|x|)\} \end{aligned}$$

respectively. Notice that for every integrand *F*, the origin of ${\mathbb {R}^{n}}$ belongs to the interior of $\text { Hypo}(F)$ and $\text { Graph}(F)\subseteq \partial (\text { Hypo}(F))$. Moreover, $K_F= \text { Hypo}(\mathcal {W}(F))$. Indeed, both sets contain the origin $0\in {\mathbb {R}^{n}}$ and, if $x\in \text { Hypo}(\mathcal {W}(F))\backslash \{0\}$, by definition of $\mathcal {W}(F)$ we have

$$\begin{aligned} |x|\le \inf _{\begin{array}{c} w\in {\mathbb {S}^{n-1}}\\ \langle x/|x|,w\rangle >0 \end{array}} \left\{ \frac{F(w)}{\langle x/|x|, w\rangle }\right\} . \end{aligned}$$

Therefore $x \in H_w$ for every $w\in {\mathbb {S}^{n-1}}$. This proves the inclusion “⊇”. To prove the other, fix a point $y\in K_F\backslash \{0\}$. Then

$$\begin{aligned} |y|\left\langle \frac{y}{|y|},w\right\rangle = \langle y , w \rangle \le F(w)\quad \forall w\in {\mathbb {S}^{n-1}}. \end{aligned}$$

Therefore,

$$\begin{aligned} |y|\le \frac{F(w)}{\langle y/|y|, w\rangle }\quad \forall w\in {\mathbb {S}^{n-1}}\text { s.t. }\langle y/|y|,w\rangle >0. \end{aligned}$$

Hence $$y\in \text { Hypo}(\mathcal {W}(F))$$.

Let $\Omega \subseteq {\mathbb {R}^{n}}$ be any bounded subset. The *support function of* Ω is the function $\beta _\Omega :{\mathbb {R}^{n}}\rightarrow {\mathbb {R}}$ given by

$$\begin{aligned} \beta _\Omega (x):= \sup _{y\in \Omega }\left\{ \langle x,y\rangle \right\} \end{aligned}$$

for every $x\in {\mathbb {R}^{n}}$. Notice that the support function of a set is always convex and 1–homogeneous. Moreover, $\beta _\Omega$ is positive (as a 1–homogeneous function) if and only if 0 is contained in the interior of Ω. A rather trivial, yet important, property of the support function is that the hyperplane $\pi _v:= \beta _\Omega (v)v + v^\perp$ is a supporting hyperplane for Ω for every $v\in {\mathbb {S}^{n-1}}$.

### Lemma 5

Let $F\in \mathbb {I}$ be an integrand.

1. $\mathcal {D}(F)$ is the support function of $K_F$. In particular, $\mathcal {D}(F)$ is a convex function and positive as a 1–homogeneous (lower semi–)continuous function. Moreover, if *G* is any other convex, 1–homogeneous positive function such that G≤F, then $G\le \mathcal {D}(F)$.
2. For every $v\in {\mathbb {S}^{n-1}}$ and $\overline{x}\in K_F$, the following are equivalent:
  - $$\mathcal {D}(F)(v)= \langle v,\overline{x}\rangle $$;
  - $$\overline{x}\in \mathcal {D}(F)(v)v +v^\perp $$;
  - $\overline{x} + v^\perp$ is a supporting hyperplane for $K_F$.
3. $$\text { Ort}(\partial K_F)\subseteq \text { Cont}(F)$$.

### Proof

(1) Fix $$v\in {\mathbb {S}^{n-1}}$$. Then

$$\begin{aligned} \mathcal {D}(F)(v) = \sup _{w\in {\mathbb {S}^{n-1}}}\left\{ \mathcal {W}(F)(w)\langle v,w\rangle \right\} = \sup _{y\in K_F}\left\{ \langle v,y\rangle \right\} = \beta _{K_F}(v). \end{aligned}$$

Since both $\mathcal {D}(F)$ and $\beta _{K_F}$ are 1–homogeneous, they coincide in ${\mathbb {R}^{n}}$. Suppose now *G* to be a convex, 1–homogeneous positive function. Then *G* is the support function of the set

$$\begin{aligned} \Omega _G:=\{y\in {\mathbb {R}^{n}}: \langle v,y\rangle \le G(v)\,\forall v\in {\mathbb {S}^{n-1}}\}. \end{aligned}$$

If G≤F, then $\Omega _G\subseteq K_F$ and, as $\mathcal {D}(F)$ is the support function of $K_F$, this implies $G\le \mathcal {D}(F)$.

(2) Fix $v\in {\mathbb {S}^{n-1}}$ and $\overline{x}\in K_F$. The equivalence of (b) and (c) is an immediate consequence of (1). Therefore, it is enough to prove that (a) holds if and only if (c) holds.

Suppose that $\overline{x} + v^\perp$ is a supporting hyperplane for $K_F$, then, by virtue of (1), $\mathcal {D}(F)(v)v = \overline{x} + w$, for some $w\in v^\perp$. Therefore, taking the scalar product of both with *v*,

$$\begin{aligned} \langle v,\overline{x}\rangle = \mathcal {D}(F)(v)\langle v,v\rangle = \mathcal {D}(F)(v). \end{aligned}$$

Viceversa, if $\mathcal {D}(F)(v) = \langle v,\overline{x}\rangle$, then, using the definition of support function,

$$\begin{aligned} \langle v,\overline{x}\rangle \ge \langle v,y\rangle \quad \forall y\in K_F. \end{aligned}$$

Thus, as $\overline{x}\in K_F$, the plane $\overline{x} + v^\perp$ is a supporting hyperplane for $K_F$.

(3) Fix $\overline{x}\in \partial K$ such that the (outer) unit normal $v:=N_{\partial K_F}(\overline{x})$ is well defined. Then $\overline{x} + v^\perp$ is a supporting hyperplane of $K_F$, thus, by (2),

$$\begin{aligned} \mathcal {D}(F)(v)= \langle \overline{x},v\rangle . \end{aligned}$$

On the other hand, $K_F$ is the intersection of the halfspaces

$$\begin{aligned} \{z\in {\mathbb {R}^{n}}: \langle z,w\rangle \le F(w)\}\quad w\in {\mathbb {S}^{n-1}}\end{aligned}$$

and *F* is lower semicontinuous. Hence, for every $y\in \partial K_F$ there exists $\overline{w}(y)\in {\mathbb {S}^{n-1}}$ such that

$$\begin{aligned} F(\overline{w}(y)) = \langle y,\overline{w}(y)\rangle . \end{aligned}$$

Observe that $F(\overline{w}(y))\overline{w}(y)+ (\overline{w}(y))^\perp$ is a supporting hyperplane passing through *y*. Since we are assuming that $\partial K_F$ admits a tangent space at $\overline{x}$, then $\overline{w}(\overline{x})= v$. This proves that $F(v) = \mathcal {D}(F)(v)$.

□

As a consequence of Lemma 5(1), an integrand $F\in \mathbb {I}$ is convex in the sense of Sect. 1 if and only if the function x↦F(x) is convex in ${\mathbb {R}^{n}}$ in the classical sense.

## Proof of Theorem 1 and further remarks

### Lemma 6

Let $O_F:=\text { Hypo}(\mathcal {I}\circ \mathcal {D}(F))$. Then $\mathfrak {P}O_F = K_F$ and $\mathfrak {P}K_F = O_F$. In particular, $O_F$ is a compact and convex subset containing 0 in its interior.

### Proof

By virtue of Lemma 4 and the fact that $K_F$ is a compact convex subset containing 0 in its interior, it is enough to show that $O_F = \mathfrak {P}K_F$. Using the definition of hypograph, *F*–crystal and of $\mathcal {D}= \mathcal {A}\circ \mathcal {W}$,

$$\begin{aligned} O_F = \left\{ x\in {\mathbb {R}^{n}}: \mathcal {D}(F)(x)\le 1\right\} = \left\{ x\in {\mathbb {R}^{n}}: \langle \mathcal {W}(F)(v)v,x\rangle \le 1 \,\,\forall v\in {\mathbb {S}^{n-1}}\right\} = \mathfrak {P}K_F. \end{aligned}$$

□

### Corollary 7

For any $$v\in {\mathbb {R}^{n}}$$, $$\mathcal {D}(F)(v) = \Vert v \Vert _F$$.

### Proof

Recall the definition of $\Vert \cdot \Vert _F$ given in the introduction. Then, by Lemma 6,

4 $$\begin{aligned} \Vert v \Vert _F = \min \left\{ \lambda \ge 0 : v\in \lambda O_F\right\} = \min \{\lambda \ge 0 : \mathcal {D}(F)(v)\le \lambda \} = \mathcal {D}(F)(v), \end{aligned}$$

for every $$v\in {\mathbb {R}^{n}}$$.

□

We are finally ready to prove Theorem 1.

### Proof of Theorem 1

Let $F\in \mathbb {I}$ and fix a curve $\gamma \in \text { AC}([0,1];{\mathbb {R}^{n}})$. Set $\widehat{v}:=\gamma (1)-\gamma (0)$ and $\overline{x}\in K_F$ such that $\Vert \widehat{v} \Vert _F=\langle \widehat{v},\overline{x} \rangle$. On the one hand, using (3) and Jensen’s inequality,

5 $$\begin{aligned} {\mathfrak {L}_F}(\gamma ) = \int _0^1 F(\dot{\gamma }(t))\,dt \ge \int _0^1 \mathcal {D}(F)(\dot{\gamma }(t))\,dt \ge \mathcal {D}(F)(\widehat{v}), \end{aligned}$$

and the two inequalities are equalities if and only if $\dot{\gamma }(t)\in \text { Cont}(F)$ for almost every t∈(0,1) and $\mathcal {D}(F)$ is linear in the image of $\dot{\gamma }$. On the other hand, by Corollary 7, $\mathcal {D}(F)$ is linear in the image of $\dot{\gamma }$ if and only if $\mathcal {D}(F)(\dot{\gamma }(t))= \langle \dot{\gamma }(t),\overline{x}\rangle$ for almost every t∈(0,1). Thus by virtue of Lemma 5(2), the two inequalities of (5) are equalities if and only if, for almost every t∈(0,1), $\dot{\gamma }(t)\in \text { Cont}(F)$ and $\overline{x} + (\dot{\gamma }(t))^\perp$ is a supporting hyperplane for $K_F$ at $\overline{x}$.

□

### Corollary 8

Let $F\in \mathbb {I}$ be a convex integrand and fix $\gamma \in \text { AC}([0,1];{\mathbb {R}^{n}})$. If γ is a reparametrization of a segment then γ is a *F*–geodesic.

### Proof

Under the standing assumptions, for every path $\gamma \in \text { AC}([0,1];{\mathbb {R}^{n}})$ of the form $\gamma (t)=x_0+\tau (t)\widehat{v}$, $\widehat{v}\in {\mathbb {R}^{n}}\backslash \{0\}$, both the inequalities in (5) are equalities. Thus, γ is a *F*–geodesic.

□

Now we exhibit a counterexample for the converse of Corollary 8, demonstrating that even when *F* is a convex integrand, not every *F*–geodesic needs to be a line segment.

### Example 9

Consider the 1–homogeneous positive function $F:{\mathbb {R}}^2\rightarrow {\mathbb {R}_{\ge 0}}$ defined as

$$\begin{aligned} F(x,y):= |x| + |y| \quad \forall (x,y)\in {\mathbb {R}}^2. \end{aligned}$$

Since *F* is a convex function, by Lemma 5(1), *F* is a convex integrand. Fix $\widehat{v}:=(1,1)$ and let $\gamma _0 \in \text { AC}([0,1];{\mathbb {R}}^2)$ be the function $\gamma _0(t):= t\widehat{v}$ for every t∈[0,1]. By virtue of Corollary 8, $\gamma _0$ is a *F*–geodesic. Therefore,

$$\begin{aligned} \min _{\begin{array}{c} \gamma (0) = (0,0)\\ \gamma (1)=(1,1) \end{array}} \left\{ {\mathfrak {L}_F}(\gamma )\right\} = {\mathfrak {L}_F}(\gamma _0) = \int _0^1 F(\dot{\gamma }_0(t))\,dt = 2. \end{aligned}$$

Let f,g:[0,1]→[0,1] be any two absolutely continuous, strictly, increasing bijective functions and let γ(t):=(f(t),g(t)) for every t∈[0,1]. Then $\gamma \in \text { AC}([0,1];{\mathbb {R}}^2)$ and satisfies γ(0)=(0,0) and γ(1)=(1,1). Moreover, by the fundamental theorem of calculus

$$\begin{aligned} {\mathfrak {L}_F}(\gamma ) = \int _0^1 (f'(t) + g'(t))\,dt = f(1)-f(0) + g(1)-g(0) = 2. \end{aligned}$$

Therefore, γ is a *F*–geodesics. This implies that there exist (infinitely many) *F*–geodesics connecting the points (0, 0) and (1, 1) that are different from (a reparametrization of) a line segment.

On the other hand, if $\rho =(\rho _1,\rho _2) \in \text { AC}([0,1];{\mathbb {R}}^2)$ is such that ρ(0)=(0,0) and ρ(1)=(0,1). Then

$$\begin{aligned} {\mathfrak {L}_F}(\rho ) = \int _0^1 |\rho '_1(t)|\,dt + \int _0^1 |\rho '_2(t)|\,dt \ge \int _0^1 |\rho _1'(t)|\,dt + 1 \ge 1, \end{aligned}$$

and the two equalities are both equalities if and only if $\rho _2$ is strictly increasing and ${\rho _1\equiv 0}$. Therefore, up to reparametrization, the line segment t↦(0,t) is the only *F*–geodesic connecting the points (0, 0) and (0, 1).

**Fig. 1:**
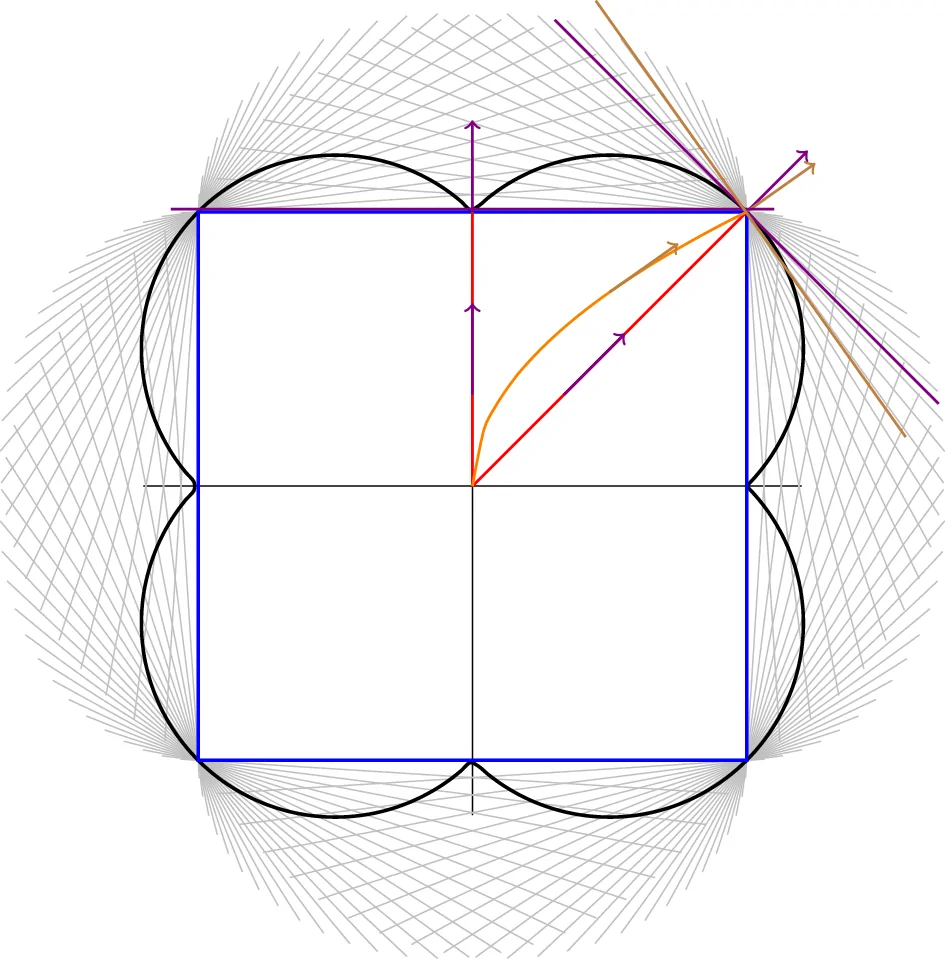
graphical representation of Example 9

A visual representation of this example is provided in Fig. 1. The thick black and blue lines represent $\text { Graph}(F)$ and the boundary of $K_F$ respectively.

## Proof of Theorem 3

Let $\Omega \subseteq {\mathbb {R}^{n}}$ be an arbitrary set. A point $x\in \Omega$ is an *extremal point of* Ω, and we write $x\in \text { Extr}(\Omega )$ if *x* cannot be written as a strictly convex combination of any two other points in Ω. Clearly, if $K\subseteq {\mathbb {R}^{n}}$ is a convex subset, then $\text { Extr}(K)\subseteq \partial K$.

The next result is well–known in the literature, and will play a key role in the sequel.

### Lemma 10

(Krein-Milman theorem) Every compact convex set in ${\mathbb {R}^{n}}$ is the convex hull of its extremal points.

### Lemma 11

Let $F\in \mathbb {I}$ be an integrand. If $\mathcal {D}(F)(v)=1$ (i.e. $v\in \partial O_F$), then $v\in \text { Ort}(\partial K_F)$ if and only if $v\in \text { Extr}(O_F)$.

### Proof

Fix $v\in {\mathbb {R}^{n}}$ such that $\mathcal {D}(F)(v)=1$. If $v\in \text { Ort}(\partial K_F)$, then exists $\overline{x}\in \partial K_F$ such that $\overline{x} + v^\perp$ is the affine tangent space of $\partial K_F$ at $\overline{x}$. Thus, there exists only one supporting hyperplane of $K_F$ at $\overline{x}$. Suppose that $\tilde{u},\tilde{w}\in O_F$ and $\lambda \in (0,1)$ are such that $v= (1-\lambda )\tilde{u} + \lambda \tilde{w}$. Then

$$\begin{aligned} 1 = \mathcal {D}(F)(v) = \langle v, \overline{x}\rangle = (1-\lambda )\langle \tilde{u},\overline{x}\rangle + \lambda \langle \tilde{w},\overline{x}\rangle . \end{aligned}$$

This implies

$$\begin{aligned} 1 =\mathcal {D}(F)(\tilde{u}) = \langle \tilde{u},\overline{x}\rangle \quad \text {and}\quad 1 =\mathcal {D}(F)(\tilde{w})= \langle \tilde{w},\overline{x}\rangle . \end{aligned}$$

Therefore, by Lemma 5(2), $\tilde{u} + \tilde{u}^\perp$ and $\tilde{w} + \tilde{w}^\perp$ are supporting hyperplanes of $K_F$ at $\overline{x}$. By uniqueness of the tangent space, $\tilde{u}=\tilde{w}=v$.

Viceversa, if $v= (1-\lambda )\tilde{u} + \lambda \tilde{w}$ for some $\tilde{u},\tilde{w}\in O_F\backslash \{v\}$ and $\lambda \in (0,1)$, then, arguing as before, one proves the existence of three different supporting hyperplanes for $K_F$ at $\overline{x}$. Therefore $v\not \in \text { Ort}(\partial K_F)$.

□

Let us introduce the following notation. If $v\in {\mathbb {R}^{n}}$, we define $\gamma _v\in \text { AC}([0,1];{\mathbb {R}^{n}})$ as $\gamma _v(t):= tv$ for every $0\le t\le 1$. The *concatenation of two paths* $\gamma ,\rho \in \text { AC}([0,1];{\mathbb {R}^{n}})$ such that $\gamma (0)=\rho (0)=0$ is the path $\gamma \diamond \rho \in \text { AC}([0,1];{\mathbb {R}^{n}})$ defined as

$$\begin{aligned} \gamma \diamond \rho (t):= {\left\{ \begin{array}{ll} \gamma (2t)& , \text { if }0\le t\le \frac{1}{2}\\ \rho (2t-1) + \gamma (1)& , \text { if }\frac{1}{2}<t\le 1 \end{array}\right. }. \end{aligned}$$

Observe that if $\gamma ,\rho ,\sigma \in \text { AC}([0,1];{\mathbb {R}^{n}})$, then the paths $\gamma \diamond (\rho \diamond \sigma )$ and $(\gamma \diamond \rho )\diamond \sigma$ differ only by a reparametrization. Therefore, with a small abuse of notation, when the choice of the parametrization is not important, we may simply write $\gamma _N\diamond \cdots \diamond \gamma _1$ for the concatenation of *N* paths such that $\gamma _j(0)=0$ for every $1\le j\le N$.

Fix a vector $v\in {\mathbb {R}^{n}}$ and suppose $v = u_1 + \cdots + u_N$ for some vectors $u_1,...,u_N\in {\mathbb {R}^{n}}$. Using the definition of ${\mathfrak {L}_{\mathcal {D}(F)}}(\cdot )$ and the 1–homogeneity of $\mathcal {D}(F)$, one immediately shows

6 $$\begin{aligned} {\mathfrak {L}_{\mathcal {D}(F)}}(\gamma _v) = \mathcal {D}(F)(v) \quad \text {and}\quad {\mathfrak {L}_{\mathcal {D}(F)}}(\gamma _{u_{N}}\diamond \cdots \diamond \gamma _{u_1}) = \sum _{j=1}^N \mathcal {D}(F)({u_j}). \end{aligned}$$

Moreover, by Lemma 5(1), Jensen’s inequality for sums and the 1–homogeneity of $\mathcal {D}(F)$ it follows that

$$\begin{aligned} \mathcal {D}(F)(v) = N\mathcal {D}(F)\left( \frac{u_1+\cdots + u_N}{N}\right) \le \sum _{j=1}^N \mathcal {D}(F)(u_j). \end{aligned}$$

Therefore,

7 $$\begin{aligned} {\mathfrak {L}_{\mathcal {D}(F)}}(\gamma _{ u_1 + \cdots + u_N}) \le {\mathfrak {L}_{\mathcal {D}(F)}}(\gamma _{u_N}\diamond \cdots \diamond \gamma _{u_1}) \quad \forall u_1,..., u_N\in {\mathbb {R}^{n}}. \end{aligned}$$

On the other hand, if there exist $\tilde{u}_1,...,\tilde{u}_N\in {\mathbb {R}^{n}}$ and $\lambda _1,...,\lambda _N\in [0,1]$ with $\lambda _1+...+\lambda _N=1$ such that

$$\begin{aligned} v = \sum _{j=1}^N \lambda _j \tilde{u}_j \quad \text {and}\quad \mathcal {D}(F)(\tilde{u}_j)=\mathcal {D}(F)(v)\,\forall 1\le j\le N, \end{aligned}$$

then, setting $u_j:= \lambda _j \tilde{u}_j$ for each $1\le j\le N$ one obtains equality in (7).

### Proof of Theorem 3

Let $F\in \mathbb {I}$ be an integrand and $x,y\in {\mathbb {R}^{n}}$ be two distinct points. Without loss of generality, suppose x=0 and $\mathcal {D}(F)(y)=1$

(1) If $y\in \text { Ort}(\partial K_F)$, then exists $\overline{x}\in \partial K_F$ such that $y/|y| = N_{\partial K_F}(\overline{x})$ is the (outer) unit normal to $\partial K_F$ at $\overline{x}$. Therefore there exists one unique supporting hyperplane for $K_F$ at $\overline{x}$ and this is $\overline{x} + y^\perp$. Applying Theorem 1, every geodesic $\gamma \in F\text { --Geo}(0,y)$ must satisfy $\dot{\gamma }(t)\in \text { Cont}(F)\cap \text { span}^+\{y\}$ for almost every t∈(0,1), where

$$\begin{aligned} \text { span}^+\{y\}:= \{\lambda y: \lambda \ge 0\}. \end{aligned}$$

Since, by Lemma 5(3) $\text { Ort}(\partial K_F)\subseteq \text { Cont}(F)$, then $\text { Cont}(F)\cap \text { span}^+\{y\}=\text { span}^+\{y\}$. In particular, γ must be a reparametrization of $\gamma _y$.

(2) Suppose now $y\not \in \text { Ort}(\partial K_F)$. Then, by Lemma 11, *y* is not extremal in $O_F$. Using Lemma 10 and Lemma 11 once again, we find $\lambda \in (0,1)$ and $\tilde{u},\tilde{w}\in \text { Ort}(\partial K_F)$ with $\mathcal {D}(F)(\tilde{u})=\mathcal {D}(F)(\tilde{w})=1$ and such that y=u+w, where $u:=(1-\lambda )\tilde{u}$ and $w:=\lambda \tilde{w}$. For any $\tau \in [0,1]$, consider the curve $\sigma ^\tau :[0,1]\rightarrow {\mathbb {R}^{n}}$ defined as

$$\begin{aligned} \sigma ^\tau (t):= \gamma _{(1-\tau )u}\diamond \gamma _{w}\diamond \gamma _{\tau u}. \end{aligned}$$

Then, for any $\tau _1\ne \tau _2$ we have that $\sigma ^{\tau _1}$, is not a reparametrization of $\sigma ^{\tau _2}$ and, by Lemma 5(3), Corollary 4 and the above remark,

8 $$\begin{aligned} {\mathfrak {L}_F}(\sigma ^\tau ) = \mathcal {D}(F)(u) + \mathcal {D}(F)(w) = \mathcal {D}(F)(y) = \Vert y \Vert _F\quad \forall \tau \in (0,1). \end{aligned}$$

As $\sigma ^\tau (0)=0$ and $\sigma ^\tau (1)=y$ for every $\tau \in [0,1]$, Theorem 1 and (8) prove that $\{\sigma ^\tau :0\le \tau \le 1\}$ is an infinite family of *F*–geodesic, each one of them identifying a different element in ${F\text { --Geo}}_{/\sim }(0,y)$. □

## Data Availability

No datasets were generated or analysed during the current study.
